# Why “Pulling” Teeth Fails

**Published:** 1920-04

**Authors:** Robert Burns


### WHY "PULLING" TEETH FAILS

### A Plea for Their Surgical Removal When Evulsion of These Organs is Indicated by Infection

BY ROBERT BURNS, JR., D.D.S.

This paper is a plea for what has come to be known as the surgical removal of teeth whenever the evulsion of these organs is indicated, either by reason of pyorrhea, abscesses, or in any condition where infection is present about the root or in the periapical tissue. An attempt will be made to prove that in the presence of the aforementioned conditions the ordinary "pulling" of teeth offers but scant hope for the entire removal of the infection. Moreover, any attempts at curetment in the sockets so remaining will, in the great majority of cases, not only absolutely fail of its purpose, but actually do more harm than good, because all the chances are against a proper removal of the focus and rather more likely to stir up the infection with the resultant exacerbation of a variety of systemic ills more or less harmful and certainly very unpleasant for the patient.

No dead or devitalized tooth should ever be merely "pulled." In every instance involving a dead tooth where for whatsoever reason removal has been decided upon, surgical removal should always be the method of choice.

These statements are based upon an acceptance of the fact that dead or devitalized teeth, abscessed teeth, or teeth badly infected with pyorrhea are in an alarmingly large percentage of cases foci of infection, the proper removal of which is a matter of very serious import. This fact has been so clearly proved by the work of Billings, Hartzell, Rosenow, and others, that it seems hardly worth while mentioning further.

One reason why so many medical men and a still greater number of dentists put too much faith in the "treatment" of root-canals for the cure of abscesses and the removal of infection, is that they fail to grasp the fact that every tooth in which the pulp has died or been removed at once becomes in every sense of the word a "dead" tooth. The dentin, being composed of about 28 per cent. organic matter from which all blood supply has been removed, can necessarily do nothing else sooner or later than become infected.

Noyes says: "The vital function of the pulp is the formation of dentin and is performed by the layer of odontoblasts. These cells also, by means of their dentinal fibrils, maintain the same relation to the dentin matrix that the bone and the cement corpuscles bear to the matrix of bone and cementum. When the pulp is removed from a tooth, its dentin becomes dead dentin in the same sense that bone in which the bone corpuscles have been killed is necrosed bone." Black and Hopewell-Smith are in accord with this statement, as, indeed, are practically all dental histologists.

Dead bone can and will form a sequestrum that may be exfoliated. Not so with dead tooth dentin, for it is closely held in place by the surrounding cementum, the vitality of which is maintained by the pericementum, whence it draws its blood supply. This, however, will not prevent, as Noyes has shown, the toxic products from the dead dentin or the live bacteria getting into the lymph channels and into the blood stream. Furthermore, if the enveloping pericementum is removed or becomes infected there is absolutely no hope of cure, either spontaneously or with the assistance of medication; for the cementum is not like bone. It has no Haversian system; it is not vascular; it can not form a sequestrum; it can not heal itself.

Arthur Black, referring to pyorrheal as well as apical involvements, says: "The suppurative detachments of the peridental membrane are in practically all cases permanent detachments, whether the detachment is at the side of the root or the apex."

Wherefore, when the cementum loses its pericemental attachment and becomes infected, the only hope is complete removal by surgical interference.

These facts are accepted as basic and beyond question by dental histologists. Upon what theory, then, is based the hope of "saving dead teeth"? And let us see if the hope has even a reasonable chance of success.

First, it is agreed that every particle of pulp tissue in the root-canal must be completely removed under aseptic care. Secondly, the ends of the dentinal tubuli must be thoroughly sealed against bacterial invasion and the root apex similarly sealed. In passing, it would be interesting to know what percentage of root-canal operations in the average dental office are performed under surgical asepsis.

If, for the sake of argument, we grant that it is physically possible to remove all debris from a capillary tube with multiple apical foramina, there still remains the impossibility of sealing the tubuli and the root apex. Dr. Weston A. Price, of the National Dental Research Department, conducted a series of experiments to determine this very point. He asserts as a result of these experiments: "It is therefore a physical impossibility to completely fill a pulp chamber with gutta-percha made plastic by any of the above solvents [referring in detail to the various solvents commonly used in dentistry for the purpose] except at the time the gutta-percha contains the solvent." His experiments further proved that immediately after the filling is placed contraction commences, due to the disappearance of the solvent, leaving a space through which bacteria have free access. Hence, he concludes: "We are using materials which do not have the properties that our needs require. While contraction begins at once, it will continue slowly for months and possibly for years. This probably explains why so many root-filled teeth which at first seem normal fail months or years after filling."

In view of these facts, then, every "dead" tooth must rest under very grave suspicion. The chances are all against it, and if we have due regard for the health of the patient, there can be no real cause for taking exception to the statement, that every devitalized tooth is a potential source of danger and, in the presence of symptoms, should be surgically removed.

The comfort of the tooth, its local condition, and its serviceability alone are by no means dependable criteria as to the possibility of its harboring infection. Nor is the radiogram of unfailing value as a diagnostic aid. Neither is the condition of the gum to be given too much weight in arriving at a diagnosis. I have seen a mouth whence an abscessed tooth had been "pulled" fifteen years before and the gum gave absolutely no indication of trouble. In this case, however, the radiogram showed a sclerosed area in the jaw-bone surrounding a rarefied spot which at operation was found to be granulating tissue. In this instance, the indications for operation were the history of the "pulled" abscessed tooth, the radiographic findings and the further fact that, notwithstanding competent physicians could find absolutely no cause, the patient persisted in reporting symptoms at rather constant intervals, such as a general feeling of malaise, lack of energy, annoying pain at the back of the neck, which sometimes jumped to the wrist. She was under weight, complexion sallow, and had been told by one physician that she was "merely neurasthenic," a very convenient, if scarcely illuminating, designation. Every symptom disappeared in due course after operation.

The outstanding fact related to infected "dead" teeth is that almost invariably the infection extends into the bone beyond the apex of the root. Practically in all cases at operation it will be found that the area involved is larger than indicated by the radiograph. Except in the cystic type these areas may assume almost any shape, very often extending at right angle to the root. They often have a series of pedicles extending at various angles. Particularly is this true in involvements of the superior laterals or bicuspids. In such cases obviously it would be impossible to remove the infected areas by merely pulling the tooth and trying to curet down the socket. It would be absolutely impossible to reach an area of infection. Repeatedly at operation, when teeth were to be removed, just as a matter of demonstration of the futility of ''pulling'' teeth, after having laid back a flap and removed the alveolar bone over the root, I have with dental forceps ''pulled'' the tooth just to see if the granuloma would come out with the tooth. Failure to do so occurs in a sufficiently large number of cases, even when the root is well exposed, to warrant the positive statement that ''pulling'' teeth in the hope of removing periapical infection is absolutely contraindicated It is unsurgical, brutal, and, at best, merely guesswork. Attempting to curet down a socket is merely scratching around in the dark. If a tooth socket extends nearly to or even into the antrum such blind attempts at curetment of a socket would be very apt to carry infection into the antrum. Not infrequently I have seen small spicules of broken-down bone lifted off the antral membrane when the gum and periosteum were retracted over teeth showing rarefied areas. ''Pulling'' such teeth and trying to remove infection by cureting such a socket, could have resulted only in forcing these spicules into the antrum.

Similar conditions are often present in proximity to the inferior dental canal, often encroaching closely upon the mental foramen. It is certainly a safer procedure in any surgical operation to have a clear field with good vision. No place else in the body would any one assuming the title of surgeon follow such brutal, haphazard and dangerous methods as does the average dentist who attempts to ''pull'' a tooth by main strength and brute force. More rapid and better healing follows the surgical removal than after ''pulling.'' Naturally, this is to be expected. By the former method we make a clean triangular incision, retract the gum and periosteum over the buccal root or roots or the labial root, as the case may be. With a chisel we carefully remove the bone and lift out the root; being careful to sponge the field dry renders it possible to properly remove as much of the infected tissue as is humanly possible. There is direct vision; no trusting to a sense of touch. All rough bone being carefully smoothed, the flap is sutured into place. Dead spaces are removed—a matter of prime importance. Impossibility of doing this is one of the most serious failures of "pulling" teeth.

Let us take for illustration an upper molar with three roots, divergent and curved, two of which show areas of rarefication at the apices. Curetment, however accurate or inaccurate, as luck may be, is bound to enlarge the area at the ends of the roots. The sockets after extraction remain open, and these must remain as dead spaces, leading down to still larger dead spaces—the curetted areas, thus violating one of the best-established principles of bone surgery. Furthermore, in a very much greater number of cases than is generally known, if the teeth are "pulled" the alveolar plate is fractured, requiring a longer or shorter period to exfoliate, keeping the wound open, the tissues sore and adding to the burden of infection. Finally, when a tooth is "pulled" the soft tissues are bound to be more or less torn and ragged in a majority of cases, I care not how careful and expert the extractor may be.

When a number of teeth are to be removed prior to making a plate, if the surgical removal method is followed, the alveolar septa removed, and the bone trimmed down smooth so that a good flap can be sutured over, the patient will be vastly more comfortable, have a smoother and better ridge left and be ready for the plate sooner by far than by the "pulling" method.

Teeth that have long been infected with pyorrhea, where the pockets are deep and the alveolar process largely gone through infection, should by no means ever merely be "pulled," no matter how loose they may be. In such cases close study of good radiograms will give an idea of the amount of infection and the destruction wrought in the bone. Very seldom, if ever indeed, will there be shown the cystic-like or granulomatous growths adherent to the soft tissue of the pocket wall. Simple extraction only by the greatest good luck would rid the field of this tissue. It is difficult to remove even when it can be seen and can be picked up. It would elude even the most vigorous and well-meant curettement of a socket, presupposing that the operator had even an inkling that it was there. Such pathological tissue, left in old sockets of the type described, is very slow in being thrown off by the natural reparative forces of the body, and there invariably results as well a much longer continued process of destruction in the bone than if all of it had been removed through proper surgical removal of the teeth; so much as that the gums and bony ridges of the patient are finally left in a very much worse condition for the retention of artificial dentures—no small factor to be considered—to say nothing of the harm of leaving in an open wound any pathological tissue that is possible of removal.

Naturally, many "dead" teeth are no doubt causing no demonstrable harm to the host thereof. A wonderful consideration must be had for the defensive and recuperative forces of nature. But such is the inherent suspicion resting against "dead" teeth, as I have just shown, that when symptoms persist that can not positively be accounted for otherwise, it is not bad practice to advise the removal of the "dead" teeth, irrespective of their appearance, serviceability and comfort to the patient, and irrespective of how "good the bone looks" in the radiograph.

In a sufficiently large number of instances, the success achieved will warrant the procedure, particularly if we make no rash promises and speak frankly with the patient. For it must be especially borne in mind that systemic involvements having their genesis about dead teeth are of slow progress; their indications are varied and not always definite in their symptomatology. Many persons are going about never thoroughly sick, scarcely ever quite well, the victims of many hours' active discomfort, to put it mildly, who unquestionably will be materially benefited by the surgical removal of their "dead" teeth.

In many such cases careful physical examination often reveals nothing of consequence. Heart, lungs, kidneys, negative; urinalysis showing practically nothing save that it has been my experience to note a preponderance of cases with an acid reaction. The blood picture may be good on the whole, and here again the principal impression to be noted is the frequency with which there is a preponderance of the small lymphocytes in an otherwise good picture. Of course, notwithstanding the most careful search, there is always to be very clearly kept in mind the possibility of any of a variety of other involvements that have evaded observation. This should always be impressed upon the patient. We should be wary of rash promises and never jump at the conclusion that merely because the patient harbors one or more "dead" teeth he is bound to be made well by their removal.

Common sense, good judgment, and careful co-ordination of the gleanings of wide experience must ever be in the forefront of the armamentarium with which these problems are to be combated. Nevertheless, the fact remains that, by reason of a habit of thought, the very indefiniteness and variety of the symptoms exhibited, and the difficulty of establishing an exact relation between cause and effect, we are prone to underestimate the dangers of "dead" teeth, and finally to treat their removal as a small matter rather than to attach thereto the same significance that the removal of dead, infected bone would demand elsewhere in the body.—*Dental Cosmos*, for March.

